# Delay in presentation of symptomatic referrals to a breast clinic: patient and system factors

**DOI:** 10.1054/bjoc.1999.0990

**Published:** 2000-01-18

**Authors:** C Nosarti, T Crayford, J V Roberts, E Elias, K McKenzie, A S David

**Affiliations:** Department of Psychological Medicine, King's College School of Medicine and Institute of Psychiatry, De Crespigny Park, 103 Denmark Hill, London, SE5 8AF, UK; Department of Public Health & Epidemiology, King's College Hospital, Bessemer Rd, London, SE5 9RS, UK; Breast Unit, King's College Hospital, Bessemer Rd, London, SE5 9RS, UK

**Keywords:** patient delay, system delay, breast symptoms, breast cancer, reasons for delaying

## Abstract

We attempted to identify factors associated with delay in presentation and assessment of women with breast symptoms who attended a London breast clinic. A total of 692 consecutive symptomatic referrals, aged 40–75 years, were studied. Patient delay, assessed prior to diagnosis, was defined as time elapsing between symptom discovery and first presentation to a medical provider. This was studied in relation to: reasons for delaying, beliefs and attitudes, socio-demographic and clinical variables, psychiatric morbidity and subsequent diagnosis. Thirty-five per cent of the cohort delayed presentation 4 weeks or more (median 13 days). The most common reason given was that they thought their symptom was not serious (odds ratio (OR) = 5.32, 95% confidence interval (CI) 3.6–8.0). Others thought their symptom would go away (OR = 3.73, 95% CI 2.2–6.4) or delayed because they were scared (OR = 4.61, 95% CI 2.1–10.0). Delay was associated with psychiatric morbidity but not age. Patients who turned out to have cancer tended to delay less (median 7 days) but not significantly. Median system delay – time between first medical consultation and first clinic visit – was 18 days. Patients who thought they had cancer and those so diagnosed were seen more promptly (median 14 days). Most factors, including socio-economic status and ethnicity were non-contributory. Beliefs about breast symptoms and their attribution are the most important factors determining when women present. Health education messages should aim to convince symptomatic women that their condition requires urgent evaluation, without engendering fear in them. © 2000 Cancer Research Campaign


*British Journal of Cancer* (2000) **82**(3), 742–748
© 2000 Cancer Research Campaign
DOI: 10.1054/ bjoc.1999.0990, available online at http://www.idealibrary.com on IDEAL®

### Delay in presentation of symptomatic referrals to a breast clinic: patient and system factors

**C Nosarti[1], T Crayford[2], JV Roberts[3], E Elias[3], K McKenzie[1] and AS David[1]**

[1]Department of Psychological Medicine, King's College School of Medicine and Institute of Psychiatry, De Crespigny Park, 103 Denmark Hill, London SE5 8AF, UK; [2]Department of Public Health & Epidemiology, King's College Hospital, Bessemer Rd, London SE5 9RS, UK; [3]Breast Unit, King's College Hospital, Bessemer Rd, London SE5 9RS, UK

**Summary** We attempted to identify factors associated with delay in presentation and assessment of women with breast symptoms who attended a London breast clinic. A total of 692 consecutive symptomatic referrals, aged 40–75 years, were studied. Patient delay, assessed prior to diagnosis, was defined as time elapsing between symptom discovery and first presentation to a medical provider. This was studied in relation to: reasons for delaying, beliefs and attitudes, socio-demographic and clinical variables, psychiatric morbidity and subsequent diagnosis. Thirty-five per cent of the cohort delayed presentation 4 weeks or more (median 13 days). The most common reason given was that they thought their symptom was not serious (odds ratio (OR) = 5.32, 95% confidence interval (CI) 3.6–8.0). Others thought their symptom would go away (OR = 3.73, 95% CI 2.2–6.4) or delayed because they were scared (OR = 4.61, 95% CI 2.1–10.0). Delay was associated with psychiatric morbidity but not age. Patients who turned out to have cancer tended to delay less (median 7 days) but not significantly. Median system delay – time between first medical consultation and first clinic visit – was 18 days. Patients who thought they had cancer and those so diagnosed were seen more promptly (median 14 days). Most factors, including socio-economic status and ethnicity were non-contributory. Beliefs about breast symptoms and their attribution are the most important factors determining when women present. Health education messages should aim to convince symptomatic women that their condition requires urgent evaluation, without engendering fear in them.
© 2000 Cancer Research Campaign

**Keywords:** patient delay; system delay; breast symptoms; breast cancer; reasons for delaying

Delay in seeking medical attention after discovery of a breast symptom is an important problem (Richards et al, 1999). The thrust of public policy is to encourage early detection and diagnosis of breast cancer in order to deliver prompt treatment and improve outcome. A meta-analysis of 12 studies on delay reported that 34.2% (range 9.9–56.0%) of subjects (total = 8781) delayed more than 3 months (Facione, 1993). Personal and social variables such as: symptom attribution (Hackett et al, 1973; Averill, 1987; Nichols, 1993), beliefs and attitudes (Antonowsky and Hartman, 1974; Greer, 1974; Timko, 1987), the nature of symptoms (Adam et al, 1980; MacArthur and Smith, 1981; Caplan, 1995), health awareness (Huguley and Brown, 1981; Darrow at al, 1987; Zervas et al, 1993), personality characteristics (Douglas and Druss, 1987; Keinan et al, 1991–92; Phelan et al, 1992) socio-demographic factors (Hackett at al, 1973; Elwood and Moorehead, 1980; Eley at al, 1994, Ramirez et al, 1999) and ethnicity (Richardson et al, 1992; Gregorio et al, 1993; Eley at al, 1994), may all determine when women present with symptoms and when treatment begins.

The majority of published studies focus on delay in presentation with breast cancer symptoms rather than all breast symptoms. However, all women with breast symptoms experience worry and distress, regardless of whether they turn out to have malignant disease (Howard and Harvey, 1998). We therefore studied comprehensively the reasons that lead to delay in presentation in all symptomatic referrals to an NHS teaching hospital breast clinic, irrespective of final diagnosis. The aim of the present investigation was to isolate the risk factors for women who tend to have long delays, and who may therefore be targeted for future intervention.

We hypothesized that 'long delayers' and 'short delayers' would have different psychosocial profiles (i.e. increased General Health Questionnaire (GHQ) scores – indicative of psychiatric morbidity, lower socio-economic status, older age and belonging to an ethnic minority group), and that these factors would also relate to system delay, in line with US data (Richardson et al, 1992; Hunter et al, 1993). We also hypothesized that fear and/or denial would be positively associated with patient delay (Darrow at al, 1987; Zervas et al, 1993; Caplan, 1995). Regarding system delay, we hypothesized that symptoms other than a lump would be associated with increased delay, whereas a cancer diagnosis would be associated with decreased delay.

### SUBJECTS AND METHODS

All 708 women referred to the Breast Clinic aged between 40 and 75 years from September 1995 to January 1997 were eligible for the study. Patients were excluded if they had cognitive impairment, had been diagnosed with breast cancer in the past 5 years, or were referred either due to a family history of breast cancer or for a second opinion or from the national breast cancer screening service. Four women declined to participate and 12 were not assessed because of logistic problems. In total, 692 (97.7%) symptomatic women were interviewed. Patients were approached by a researcher in the waiting room before their first specialist consultation. Informed consent was obtained from participants.

*Received 21 June 1999*
*Received 12 August 1999*
*Accepted 10 September 1999*

*Correspondence to:* AS David

We devised and piloted a detailed semi-structured interview to measure delay, presenting symptom(s), mammogram history, previous breast problems, reasons for delay, beliefs and attitudes to cancer. Patient delay was operationally defined as the time elapsing between symptom self-discovery and the first presentation to a medical provider to seek evaluation. Care was taken to avoid encouraging retrospective falsification in estimates of delay, which might be induced by signalling disapproval or conveying any expectation on when presentation was expected. Subjects were helped to date symptom onset with specific memory probes and anchor points. System delay was defined as the time between a woman's first medical evaluation of self-discovered breast symptoms and first consultation with a breast specialist, and was assessed by recording dates of first presentation in primary care and time for referral to reach the Breast Clinic. Dates were verified using general practitioner (GP) and hospital records. The General Health Questionnaire (GHQ-12) (Goldberg and Williams, 1988) was used to measure psychiatric morbidity and the 'Health Awareness Assessment', designed and piloted for this study, elicited patients' intention to seek medical consultation for a range of ten different physical symptoms including a breast lump, persistent headache, coughing up blood, irregular periods. Clinical staff used a short semi-structured interview to collate patients' full medical history and risk factors for breast cancer.

Social and demographic data included marital status, religion, ethnicity (by self-definition), occupation, years of education and educational attainment. Information about appropriate utilization of mammographic screening services was collected, as was failure to keep clinic appointments for the current episode, GP attendance in the previous year and preference for a female consultant.

### RESULTS

### Sample characteristics

The characteristics of the sample are shown in Table 1. Eighty-seven (12.6%) women had a final diagnosis of breast cancer.

Overall, 421 women (60.8% of whole sample) presented with a breast lump with or without pain, 62 of whom were later diagnosed with breast cancer ($\chi^2 = 4.54$, $P = 0.03$). Breast pain, with or without another associated breast symptom occurred in a similar proportion of patients irrespective of final diagnosis. Other symptoms such as 'tenderness', 'thickening', nipple discharge, skin rashes etc., occurred more frequently in the non-cancer group (see Table 1).

Women were regarded as having missed a mammogram if, according to their age (i.e. 50–64 years) they would have been expected to have been invited to participate in the National Screening Programme. This percentage was significantly higher for breast cancer patients when compared with women with other breast symptoms. Only 21 women failed to keep clinic appointments for the current episode (one of whom turned out to have cancer).

### Distribution of patient and system delay

Results are given in Table 2. For the total sample ($n$ = 692) median patient delay was 13 days. The range was from 0 to 10 958 days. The distribution was highly skewed with a few subjects showing extreme delay hence logarithmic transformation was undertaken for presentation and statistical analyses (Figure 1). Patients who

**Table 1** Characteristics of breast cancer patients and those with other breast symptoms

| Variable | Cancer ($n$ =87) | Benign ($n$ = 605) | All ($n$ = 692) | Statistics |
|---|---|---|---|---|
| Mean age (years) at symptom discovery | 60.0 ± 10.5 (Median 63) | 50.7 ± 9.1 (Median 48) | 51.8 ± 9.8 (Median 49) | Z = 7.4, $P$ < 0.001[c] |
| Presenting symptom(s) | | | | $\chi^2 = 48.2$, $P < 0.001$[b] |
| Painful breast lump | 18 (20.7%) | 115 (19.0%) | 133 (19.2%) | |
| Painless breast lump | 35 (40.2%) | 178 (29.4%) | 213 (30.8%) | |
| Breast lump + other sx | 9 (10.3%) | 66 (10.9%) | 75 (10.8%) | |
| Other breast symptoms | 25 (28.7%) | 246 (40.7%) | 271 (39.2%) | |
| Socio-economic status[a] | | | | $\chi^2 = 5.7$, $P = 0.2$[b] |
| I | 3 (3.4%) | 41 (6.8%) | 44 (6.4%) | |
| II | 13 (14.9%) | 132 (21.8%) | 145 (21.0%) | |
| III | 25 (28.7%) | 184 (30.4%) | 209 (30.2%) | |
| IV | 20 (23.0%) | 113 (18.7%) | 133 (19.2%) | |
| V | 26 (29.9%) | 135 (22.3%) | 161 (23.3%) | |
| Marital status | | | | $\chi^2 = 12.6$, $P = 0.01$[b] |
| Single | 9 (10.3%) | 101 (16.7%) | 110 (15.9%) | |
| Married/remarried | 49 (56.3%) | 333 (55.0%) | 382 (55.2%) | |
| Divorced | 13 (14.9%) | 124 (20.5%) | 137 (19.8%) | |
| Widowed | 16 (18.4%) | 47 (7.8%) | 63 (9.1%) | |
| Ethnicity | | | | $\chi^2 = 17.5$, $P = 0.004$[b] |
| British | 65 (74.7%) | 335 (55.4%) | 400 (57.8%) | |
| Other White | 9 (10.3%) | 99 (16.4%) | 108 (15.6%) | |
| African | 1 (1.1%) | 42 (6.9%) | 43 (6.2%) | |
| Afro-Caribbean | 6 (6.9%) | 99 (16.4%) | 105 (15.2%) | |
| Asian | 6 (6.9%) | 30 (5.0%) | 36 (5.2%) | |
| Family history of breast cancer | 11 (16.7%) | 68 (18.2%) | 98 (18.7%) | $\chi^2$ = NS |
| No. of GP consultations in previous year | 3.5 ± 0.9 | 3.3 ± 0.8 | 3.4 ± 0.8 | Z = 1.3, $P$ = 0.2[c] |
| Mammogram | 137 (22.6%) | 31 (35.6%) | 168 (24.3%) | $\chi^2 = 6.9$, $P = 0.01$ |

Numbers in parentheses are column percentages. [a]Standard Occupational Classification (SOC). [b]Test for heterogeneity. [c]Wilcoxon rank sums test.

© 2000 Cancer Research Campaign 

**Table 2** Patient and system delay

| Variable | Cancer (*n* = 87) | Benign (*n* = 605) | All (*n* = 692) | Statistics |
|---|---|---|---|---|
| Patient delay (days) | | | | |
| Mean (±SD) | 80 ± 172 | 129 ± 700 | 122.9 ± 658 | |
| Median | 7 | 14 | 13 | Z = 0.88, *P* = 0.38 |
| System delay (days) | | | | |
| Mean (±SD) | 22.9 ± 50 | 48.2 ± 180 | 44.8 ± 170 | |
| Median | 14 | 18 | 18 | Z = 3.46, *P* = 0.0005 |

Wilcoxon rank sums test: cancer vs benign.

were subsequently diagnosed as suffering from malignant disease showed a slight non-significant tendency to present earlier than those whose symptoms were benign. When extreme delay was considered, 5.0% of patients with benign breast symptoms versus 9.2% of cancer patients delayed presentation over 1 year ($\chi^2$ = 8.5, df = 3, *P* = 0.04).

Regarding system delay, the majority of patients were seen in the clinic within 2–4 weeks, but others were sent away with no further investigation or referral; median 18 days, range was from 0 to 2889 days (8.1 years) (Figure 2 and Table 2). Patients with breast symptoms of a benign nature experienced significantly greater system delay. Cancer patients were twice as likely to see a breast specialist within three weeks of contact with their GP than patients who had benign breast symptoms, suggesting that GPs are more alert to certain symptoms and signs that were later shown to be caused by malignancy (24 (27.6%) vs 237 (39.2%); $\chi^2$ = 5.4, df = 1, *P* = 0.02). There was no correlation between patient and system delay (Kendall's Tau: *r* = 0.05).

### Long patient delay analysis

For statistical analyses, patients were divided into 'long' and 'short' delayers. Long delayers were those presenting >27 days from symptom discovery. The cut-off point of 27 days used to define delay in this study was prompted by the preferred statistical analytic technique to explore the data (logistic regression). Only 17% of patients in the present study delayed over 3 months, compared to 35% delaying for 4 weeks or more. Using the larger group maximizes the chances of reliably explaining the observed variation in delay.

### Presenting symptom and delay

Women's presenting symptoms were investigated in relation to short and long presentation delay, and results of an analysis of variance showed no significant differences (F = 0.10, df = 4, *P* = 0.4). However, when presenting symptom was simply divided into lump/no lump and pain/no pain, statistically significant differences in delay emerged. Sixty-six per cent of women with a breast lump presented to their doctor within 27 days from symptom discovery, compared to 34% of those without a lump ($\chi^2$ = 13.9, df = 1, *P* = 0.001). Of patients with breast tenderness or pain, 76% presented to their doctor within 27 days from symptom discovery, compared to 62% of those without pain ($\chi^2$ = 8.8, df = 1, *P* = 0.003).

### Reasons for delay

### *Whole sample*

Reasons given by patients to justify their delay are given in Table 3. When patients mentioned more than one reason, we identified the most important by further questioning. Over half of 'long delays' occurred in women who believed that their symptom was not serious and therefore did not require medical attention. Women

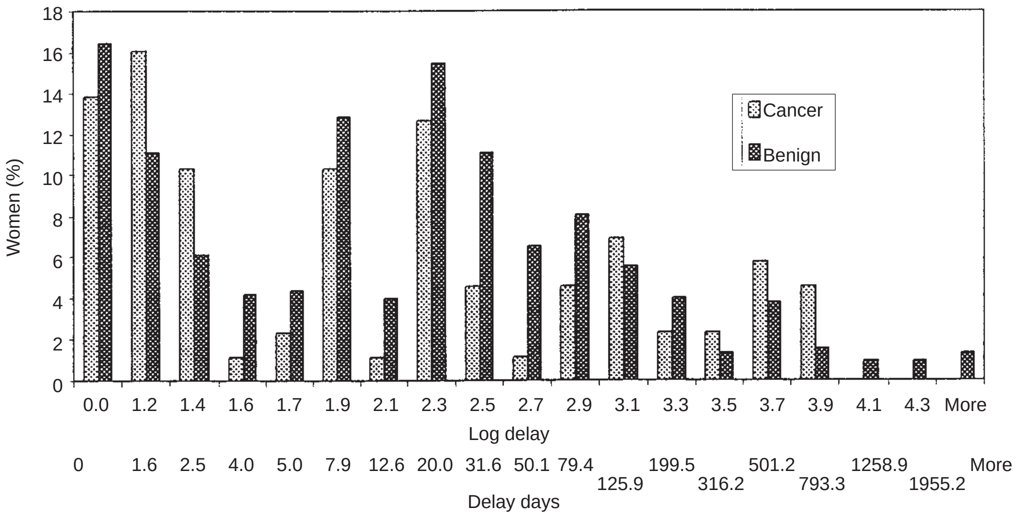

**Figure 1** Distribution of log patient delay (days) +1 for cancer patients and those with other breast symptoms

*© 2000 Cancer Research Campaign*

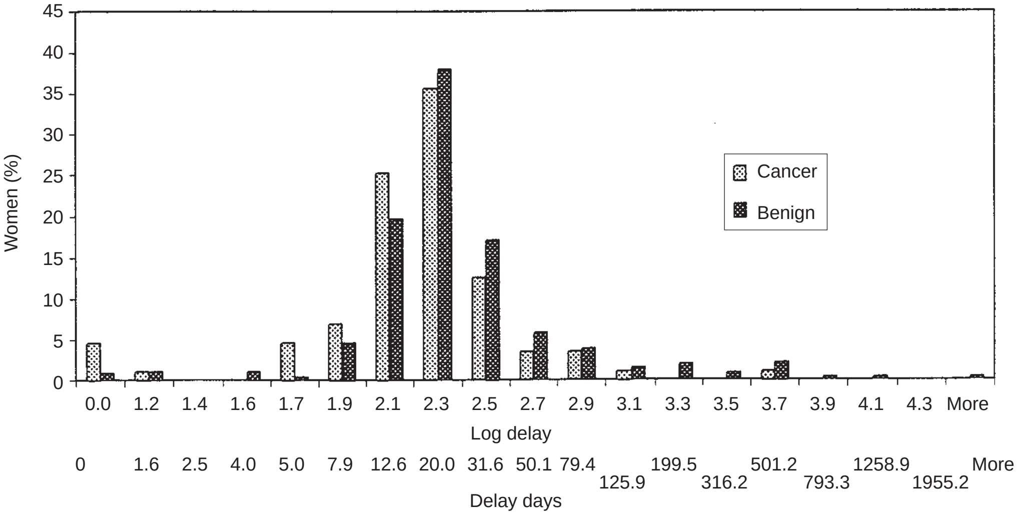

**Figure 2** Distribution of log system delay (days) +1 for cancer patients and those with other breast symptoms

**Table 3** Reasons for patient delay for the whole sample

| Reason for Delay | Frequency | Mean delay (days) and s.d. | Median delay (days) | Delay range (days) |
|---|---|---|---|---|
| Thought the symptom would go away | 85 (12.3%) | 127 ± 434 | 26 | 1–3836 |
| Thought the symptom was not serious | 231 (33.4%) | 197 ± 798 | 31 | 0–9486 |
| Scared | 34 (4.9%) | 102 ± 191 | 26.5 | 0–762 |
| Didn’t have time to seek help | 25 (3.6%) | 29 ± 40 | 14 | 0–123 |
| Other reasons | 317 (48.8%) | 77 ± 648 | 2 | 0–10958 |
| Total | 692 (100.0%) | 267 | 80.2 | |

who delayed specifically because they feared a cancer diagnosis had the highest median delay (91 days). When a reason for delay was not given and when women presented as soon as possible, delay reason was coded as ‘other’.

### *Cancer vs non-cancer*

Overall, cancer patients and patients with benign breast disease differed significantly in their reasons for delay ($\chi^2 = 13.3$, df = 4, $P = 0.01$): patients who subsequently were diagnosed with cancer delayed presentation because they were scared more often than women with benign conditions (11.5% and 4.0% respectively) and were less likely to consider their symptom to be ‘not serious’ (21.8% vs 35.0%).

When ‘long delayers’ only were considered, cancer patients delayed presentation because they were scared more often than women with benign disease (22.2% and 5.1% respectively: $\chi^2 = 10.8$, df = 1, $P = 0.001$) (Table 4). There was no significant difference between the groups in terms of thinking that the symptom would go away ($\chi^2 = 1.6$, df = 1, $P = 0.2$) or other beliefs. These results suggest that some women anticipated their eventual diagnosis of breast cancer. We examined this further by cross-tabulating attribution (benign/malignant) by diagnosis, for all subjects. Of people with benign breast symptoms, 108 (17.8%) thought they had cancer. Of those 87 women with cancer, 55 (63.2%) suspected a correct diagnosis; 32 (36.8%) thought they had a less serious condition ($\chi^2 = 16.9$, df = 1, $P = 0.001$).

In order to investigate psychosocial and demographic variables associated significantly with long patient delay (log patient delay (days) + 1), a logistic regression model was constructed. The results and significant variables are shown in Table 5. ‘Long delayers’ were characterized by poor health awareness about hypothetical breast symptoms, the tendency to minimize the significance of their symptom and by fear and higher levels of psychological morbidity (GHQ-12 scores). Variables entered which did not contribute noticeably to the variance included marital status, social class, ethnicity, educational attainment, psychiatric history, history of benign breast symptoms, family history and previous mammograms. Patient delay in cancer patients and those with benign breast symptoms was predicted by the same variables, therefore a breakdown according to diagnosis is not given.

As argued above, a cut-off of 27 days was used to define long delay. However, to aid comparison with other studies the same analysis was conducted with a 90-day cut-off. The maximum re-scaled $R^2$ was 0.21. The same explanatory variables emerged but with additions, namely preferring a female consultant: odds ratio (OR) 2.16 (95% confidence intervals (CI) 1.3–3.5); having a recent GP consultation (within 1 year of current episode): OR 1.35 (95% CI 1.03–1.78); and presenting with a lump: OR 0.56 (95% CI 0.36–0.89).

A linear regression model was constructed to predict changes in log system delay (Table 6). Women with longer patient delays and

© 2000 Cancer Research Campaign

**Table 4** Reasons for patient delay for long delayers only (>27 days), by diagnosis

| Reason for delay | Frequency | |
|---|---|---|
| | Benign | Cancer |
| Thought the symptom would go away | 35 (16.2%) | 7 (25.9%) |
| Thought the symptom was not serious | 116 (53.7%) | 11 (40.7%) |
| Scared | 11 (5.1%) | 6 (22.2%) |
| Didn't have time to seek help | 6 (2.8%) | 1 (3.7%) |
| Other reasons | 48 (22.2%) | 2 (7.4%) |
| Total | 216 (88.9%) | 27 (11.1%) |

$\chi^2$ for heterogeneity = 14.78, df = 4, *P* = 0.005.

**Table 5** Modelling patient delay (>27 days) for the whole sample, using logistic regression analysis.

| Variable | Parameter estimate | $P\,\chi^2$ | Odds ratio | 95% Confidence intervals |
|---|---|---|---|---|
| Increase in GHQ score of one unit | 0.04 | 0.03 | 1.04 | 1.00–1.09 |
| Would tend to delay presentation with physical symptoms | 0.03 | 0.002 | 1.03 | 1.01–1.05 |
| Delayed because she thought the symptom would go away | 1.44 | 0.001 | 4.23 | 2.50–7.20 |
| Delayed because she thought the symptom not serious | 1.81 | 0.001 | 6.10 | 4.11–9.17 |
| Delayed because she was scared | 1.57 | 0.0001 | 4.79 | 2.25–10.24 |
| Lump was a presenting symptom | –0.33 | 0.09 | 0.72 | 0.49–1.06 |
| Pain was a presenting symptom | –0.46 | 0.08 | 0.63 | 0.38–1.06 |

$R^2$ = 0.18, Max-rescaled $R^2$ = 0.25.

**Table 6** Modelling system delay for the whole sample using multiple regression analysis.

| Variable | Parameter estimate | $P\,\chi^2$ | % contribution to $R^2$ |
|---|---|---|---|
| Increase of 1 in log (patient delay (days) + 1) | 0.05 | 0.016 | 8.8 |
| Did not attend outpatient appointment | 0.40 | 0.0001 | 29.0 |
| Turned out to have cancer | –0.13 | 0.006 | 18.2 |
| Thought symptom was cancerous | –0.08 | 0.039 | 11.6 |
| Had a breast lump at the time of referral to the clinic | –0.09 | 0.01 | 10.0 |

$R^2$ = 0.11.

those who failed to keep their appointment tended to be processed more slowly by the system. Predictors of decreased system delay included subsequent cancer diagnosis, patients' attribution of cancer to their symptom and presence of a breast lump at the time of referral. Other socio-demographic factors including age, socio-economic status and ethnicity, were not important predictors.

We investigated the variables predictive of a positive cancer diagnosis using logistic regression ($R^2$ = 0.36). After correcting for age and demographic variables, the main results were that women later diagnosed with breast cancer were more likely to have a delayed presentation due to fear than those with benign disease, OR 2.69 (95% CI 0.98–6.9), to have thought their symptom was due to cancer, OR 2.50 (95% CI 1.4–4.6), and were significantly less likely to have had a screening mammogram OR 2.28 (95% CI 1.06–5.0).

### DISCUSSION

### Patient delay

Based on a large consecutive series of symptomatic women attending a hospital breast clinic, we found that psychological rather than demographic factors (Antonowsky and Hartman, 1974; Williams et al, 1976; Burgess et al, 1998) were the main predictors of delay in presentation of 4 weeks or more. Ethnicity did not significantly influence either patient or system delay, in contrast to reports from the USA (Richardson et al, 1992; Hunter et al, 1993), and marital status was not found to influence delay (see also Ramirez et al, 1999).

Others have taken 3 months as the criterion for long delay. Relatively few patients in this and another recent UK survey (Burgess et al, 1998) delayed for this long, moreover, the factors

 *© 2000 Cancer Research Campaign*

related to 4 weeks or more delay were also related to delay of 3 months. Additional factors emerged with longer delays such as preference for a female consultant, also highlighted in relation to attitudes to breast examination (Haigney et al, 1997). Another was having had a recent consultation with the GP, which may have inhibited patients from 'bothering' the doctor again.

Psychological distress – as indexed by an expression of fear of cancer and by GHQ scores – was associated with long delays, especially in those who did indeed turn out to have breast cancer. Not unexpectedly, those least anxious about their symptoms also delayed seeking medical attention. This pattern is predicted by an arousal model of human behaviour (Tones, 1980). Anxiety and depression were assessed at interview, so may also have coloured explanations given for previous behaviour and may be different from that at the time of symptom discovery. Similarly, patients' tendency to procrastinate when faced with the hypothetical scenario of discovering a breast lump, was inferred retrospectively with respect to patient delay, from the Health Awareness Assessment.

Health education campaigns state that a breast lump is a possible indicator of breast cancer. The proportion of women actually presenting with such a symptom in this study was 61%. Women with lumps had less patient and system delay, supporting our hypothesis and previous studies (MacArthur and Smith, 1981; Gould-Martin et al, 1982).

Nine out of 10 women (497/529) attending the clinic thinking they had a benign condition were correct, and two out of three women (108/163) who suspected they had cancer were incorrect. Nevertheless, women later diagnosed with breast cancer seemed to have a strong inkling that this was the case prior to definitive tissue diagnosis; they were interviewed after having seen a primary care practitioner, who may have cued them to the suspicion of malignancy. Despite this and contrary to previous studies (Greer, 1974) most of the women in this study subsequently diagnosed with breast cancer did not present earlier than those with a benign condition and a few delayed presentation to a worrying degree, citing fear of diagnosis as an explanation. Overall, the results suggest that factors causing delay are the same in those who develop cancer and those who do not.

A relationship between delay in symptomatic presentation and compliance with mammographic screening might have been expected since the psychological processes involved appear to overlap (Fink, 1977). Our data on mammograms were based entirely on self reports so may not have been reliable. However, no such relationship was detected. Some women claimed not to have received an offer of screening and this may have been the result of administrative failures. The current study was restricted to those who had symptoms although it covered an age range which included that which is targeted for screening (50–64 years). Caution is needed before extrapolating our findings to women diagnosed through screening or other age groups; indeed restricting our sample to those of 75 years of age or less may have obscured a relationship between age and delay found in a recent systematic review (Ramirez et al, 1999). Furthermore, differences in patient (and system) delay in other groups, such as those with and without a positive family history were not detected.

### *System delay*

This was not predicted by socio-demographic variables and our model succeeded in explaining only 11% of the variance. System delay was predicted by increased patient delay and missed appointments. Failure to attend an outpatient appointment may, however, be due in part to administrative complications, such as incorrect addresses. Further audit of this is necessary. The system we studied did not show a social class, ethnic or education bias. Furthermore, apart from a few unfortunate exceptions where patients appeared to have been falsely reassured, i.e. not referred on immediately, the system responded appropriately more quickly to patients who turned out to have cancer.

What are the implications from this study for health promotion? There is a delicate balance between scaring women away from their doctors on the one hand (especially when they suspect the worst), and adding to a false sense of reassurance, thus taking away any motivation to come forward. Women should be encouraged to think of their breast symptom as 'urgent but not necessarily serious' and should be prompted to present to their doctor as soon as possible. Diagnosis should be cast in as positive a light as possible. Although breast lumps are the most common symptom, others are also predictive of cancer and perhaps these should be mentioned in health awareness campaigns. Regarding system delay, administrative arrangements should be regularly monitored in terms of national targets. The availability of female consultants should be increased to meet patient preferences. Finally, health care professionals should be alert to women who are hesitant to present, and in such circumstances should themselves act swiftly.

### ACKNOWLEDGEMENTS

We thank all the patients and staff from the Breast Clinic for their co-operation. The King's Fund, the Department of Health and a King's Medical Research Trust Studentship (awarded to Chiara Nosarti), supported this study.

### REFERENCES

Adam SA, Horner, JK and Vessey MP (1980) Delay in treatment for breast cancer. *Commun Med* **2:** 195–202

Antonowsky A and Hartman H (1974) Delay in the detection of cancer: a review of the literature. *Health Educ Monogr* **2**: 98–128

Averill JR (1987) Emotion and psychological defense. In: *Taking Care: Understanding and Encouraging Self-protective Behaviour*, Weinstein ND (ed), pp. 54–78. Cambridge University Press: Cambridge

Burgess CC, Ramirez AJ, Richards MA and Love SB (1998) Who and what influences delayed presentation in breast cancer? *Br J Cancer* **77**: 1343–1348

Caplan LS (1995) Patient delay in seeking help for potential breast cancer. *Public Health Rev* **23**: 263–274

Darrow SL, Schoenfeld ER, Cummings KM, Wilkes E and Madoff S (1987) Women's knowledge and beliefs about breast cancer risk factors, symptoms, detection methods, and treatments. *J Cancer Educ* **2**: 165–176

Douglas CJ and Druss RG (1987) Denial of illness: a reappraisal. *Gen Hosp Psychiatry* **9**: 53–57.

Eley JW, Hil HA, Chen VW, Hunter CP and Herman AA (1994) Racial differences in survival from breast cancer. Results of the National Cancer Institute Black/White Cancer Survival Study. *JAMA* **272**: 947–954

Elwood JM and Moorehead WP (1980) Delay in diagnosis and long-term survival in breast cancer. *Br Med J* **280**: 1291–1294

Facione N (1993) Delay vs. help seeking for breast cancer symptoms: a critical review of the literature on patient and provider delay. *Soc Sci Med* **36**: 1521–1534

Fink R (1977) Delay behavior in breast cancer screening. In: *Cancer: The Behavioral Dimensions*, Cullen JW, Fox BH and Isom RW (eds), pp. 23–33. Raven: New York

Goldberg D and Williams P (1988) *A User's Guide to the General Health Questionnaire*. NFER Nelson: Windsor

© 2000 Cancer Research Campaign

Gould-Martin D, Paganini-Hill A, Casagrande C, Mack T and Ross RK (1982) Behavioural and biological determinants of surgical stage of breast cancer. *Prev Med* **11**: 429–440

Greer S (1974) Psychological aspects: delay in the treatment of breast cancer. *Proc R Soc Med* **67**: 470–473

Gregorio DI, Cummings KM and Michalek A (1983) Delay, stage of disease, and survival among White and Black women with breast cancer. *Am J Public Health* **73**: 590–593

Hackett TP, Cassem NH and Raker JW (1973) Patient delay in cancer. *New Engl J Med* **289**: 14–20

Haigney E, Morgan R, King D and Spencer B (1997) Breast examinations in older women: questionnaire survey of attitudes of patients and doctors. *Br Med J* **315**: 1058–1059

Howard RA and Harvey PG (1998) A longitudinal study of psychological distress in women with breast symptoms. *J Health Psychol* **3**: 215–226

Huguley CM and Brown RL (1981) The value of breast self-examination. *Cancer* **47**: 989–995

Hunter CP, Redmond CK, Chen VW, Dignam JJ, Edwards BK and Shapiro S (1993) Breast cancer: factors associated with stage at diagnosis in black and white women. Black/White Cancer Survival Study Group. *J Natl Cancer Inst* **85**: 1129–1137

Keinan G, Carmil D and Rieck M (1991–1992) Predicting women's delay in seeking care after discovery of a lump in the breast: the role of personality and behavioural patterns. *Beh Med* Winter: 177–183

MacArthur C and Smith A (1981) Delay in breast cancer and the nature of presenting symptoms. *Lancet* **1**: 601–603

Nichols S (1983) Reluctance to seek medical advice about breast symptoms. *J R Coll Gen Pract* **33**: 163–166

Phelan M, Dobbs J and David AS (1992). 'I thought it would go away': patient denial in breast cancer. *J R Soc Med* **85**: 206–207

Ramirez AJ, Westcombe AM, Burgess CC, Sutton S, Littlejohns P and Richards MA (1999) Factors predicting delayed presentation of symptomatic breast cancer: a systematic review. *Lancet* **353**: 1127–1131

Richards MA, Westcombe AM, Love SB, Littlejohns P and Ramirez AJ (1999) Influence of delay on survival in patients with breast cancer: a systematic review. *The Lancet* **353**: 1119–1126

Richardson JL, Langholz B, Bernstein L, Burciaga C, Danley K and Ross RK (1992) Stage and delay in breast cancer diagnosis by race, socioeconomic status, age and year. *Br J Cancer* **65**: 922–926

Timko C (1987) Seeking medical care for a breast cancer symptom: determinants of intentions to engage in prompt or delay behavior. *Health Psychol* **6**: 305–328

Tones BK (1980) Review of symposium. In: *Communication and Cancer Education*, Deeley TJ (ed). Tenovus Cancer Information Centre: Cardiff

Williams EM, Baum M and Hughes LE (1976) Delay in presentation of women with breast disease. *Clin Oncol* **2**: 327–331

Zervas IM, Augustine A and Fricchione GL (1993) Patient delay in cancer. A view from the crisis model. *Gen Hosp Psychiatry* **15**: 9–13

*© 2000 Cancer Research Campaign*
